# Depression stigma and management of suicidal callers: a cross-sectional survey of crisis hotline counselors

**DOI:** 10.1186/s12888-019-2325-y

**Published:** 2019-11-06

**Authors:** Ines Heinz, Roland Mergl, Ulrich Hegerl, Christine Rummel-Kluge, Elisabeth Kohls

**Affiliations:** 1 Department of Psychiatry and Psychotherapy, Medical Faculty, University Leipzig, Semmelweisstr. 10, Haus 13, 04103 Leipzig, Germany; 2 German Alliance Against Depression, Goerdelerring 9, 04109 Leipzig, Germany; 3 Bundeswehr University Munich, Institute of Clinical Psychology and Psychotherapy, Werner-Heisenberg-Weg 39, 85577 Neubiberg, Germany; 4 Department of Psychiatry, Psychosomatic Medicine and Psychotherapy, University Hospital Frankfurt, Goethe University Frankfurt (Distinguished Professorship funded by Dr. Senckenbergische Stiftung), Heinrich-Hoffmann-Strasse 10, 60528 Frankfurt am Main, Germany

**Keywords:** Depression stigma, Suicide prevention, Helpline, Crisis hotline, Counselor

## Abstract

**Background:**

Crisis hotlines play a key role in suicide prevention worldwide following different approaches regarding risk assessment and management of suicidality. This is to our knowledge the first study investigating depression stigma in crisis hotline counselors. The association between stigma and self-rated knowledge and their exploration of suicide risk and consecutive management of suicidal callers is being investigated.

**Methods:**

Data on depression stigma, self-rated knowledge, self-reported exploration and management of suicidality was collected from 893 counselors working for the German crisis hotline. Stigma in counselors had been compared to matched population sample (1002).

**Results:**

Crisis hotline counselors reported significantly lower depression stigma compared to the general population. Depression stigma and age associations differed in both samples. The reported exploration of suicide risk in callers differed depending on the self-rated knowledge about suicidality and depending on the personal depression stigma, but not the reported consecutive management.

**Conclusion:**

Compared to the general population, crisis hotline counselors seem to have fewer stigmatizing attitudes toward depression. Attitudes and self-rated knowledge seem to influence the confidence in counselors regarding the exploration of suicidal callers, but not the consecutive management. The results indicate that a profound training and hands-on information about depression and suicide risk seem to be essential.

## Background

Crisis hotlines play a key role in suicide prevention efforts worldwide [1–3]. They provide an important contact point for people in suicidal or emotional crisis state allowing callers to talk anonymously and without fearing stigma or discrimination [4]. Moreover, hotline counselors can contribute to decrease stigmatization, e.g. by addressing callers´ unfavorable perceptions of mental health problems and seeking professional help, which are among the most prevalent barriers to mental health care [1].

Stigma of mental health professionals toward mental illnesses, in particular depression, and in comparison to the general public has been intensively investigated (see [5]) showing inconsistent results [5, 6]. The majority of publications report no differences in beliefs about mental illnesses between mental health care providers and the population, or even less favorable ones hold by professionals [6]. Especially in the desire for social distance as an aspect of stigmatization, professionals do not differ from lay people or even show a greater social distance [6, 7] depending on the disorder e.g. being higher for schizophrenia than depression (e.g. [8, 9]). An aspect that is discussed when comparing mental health professionals’ attitudes with attitudes of the general population is the role of personal contact to people with mental illnesses [5]. A professional contact, especially with patients with severe chronic illnesses is discussed to have a different effect on attitudes and stigma, than a social contact, e.g. with family members or other personal experiences. In line with social contacts a lower personal depression stigma is reported [10, 11]. When interpreting the result of these studies, some methodological aspects have to be taken into account like using different methods for measuring attitudes as well as self-report measures instead of assessing actual behavior. Moreover, a potentially lower tendency to answer socially desirable of mental health professionals is also discussed [5].

Nevertheless, stigmatizing attitudes of mental health professionals have an impact on the health care of affected persons in different ways and are reflected by e.g. insufficient information on diagnosis and treatment, therapeutic pessimism regarding prognosis and treatment outcomes as well as discriminatory behavior (e.g. avoidance and rejection of a patient, see [6]). There is some evidence, that these factors are associated with the course of treatment and treatment adherence, which has mainly been investigated for psychotic and borderline personality disorder (see [5, 6, 12]). Mental health related stigma also affects the treatment of physical illnesses for patients with a psychiatric diagnosis and is associated with fewer medical services, lower quality of and delays in treatment, e.g. due to less referrals to specialist care [5].

Crisis hotline counselors may function as gatekeeper to mental health care for callers with a psychiatric diagnose as well as for callers with mental pain and in need of professional treatment [13]. There are recent clinical suggestions to consider suicide as complex and not always involving features of a psychiatric disorder [13].

To our knowledge there is recently no study addressing depression stigma of crisis hotline counselors and its association with the exploration of suicide risk and the subsequent management of suicidal callers.

Currently, there are different policies and approaches being applied by crisis hotline providers, e.g. hotlines in the USA (certified by the American Association of Suicidology) follow a collaborative problem-solving approach, which also includes initiating active rescue activities in collaboration with emergency services, even if a callers´ confidentiality is infringed [14–17]. Other crisis hotlines, e.g. following the Samaritan movement, focus on non-directive and active listening, and empowerment of the caller. Respecting the principle of anonymity and secrecy as well as everyone’s principal right to decide to die is of great importance within this approach. In case of imminent suicide risk, intervening against the caller’s wish is therefore not consistently mandatory [14, 16, 18–20].

In the literature there are some results indicating short-term and intermediate effects, e.g. an improvement of the callers´ mental state [3, 21] and a significant decrease in suicide status during the call [21–24]. In sum, the evidence is to be considered as limited, since these studies were uncontrolled. Investigating long-term outcome of crisis hotlines on changes in suicide rates within a population is methodologically challenging [17, 22]. Nevertheless, ecological and time series analysis have been performed comparing areas with and without crisis services or areas before and after establishing those services covering observational periods between 5 and 20 years. Some of them indicate a preventive, albeit not consistently significant, effect [25–27].

The main crisis hotline in Germany is the Telephone Emergency Service (Telefonseelsorge, TES) providing free-of-charge availability 24/7 answering approximately 1.8 million calls per year, whereas one third of the callers report a psychiatric diagnosis [28]. Further, in approximately 50,000 calls per year suicidality is of reason [29]. This is in line with numbers reported in other international studies [1, 17, 22, 23, 30, 31] . German TES counselors receive training and regular supervision, but the extent to which the mandatory trainings contain information on mental health, exploring suicide risk and managing potential suicidal callers varies between the local service centers (B. Bloemeke, personal communication, July 26, 2017). All TES centers share the principles of anonymity and non-directive listening and there is neither an obligation for the counselor to initiate rescue activities, nor is a standardized risk assessment of suicidality required.

### Aims and hypotheses

This study is the first to our knowledge investigating:

1. Depression stigma in TES counselors and compared to an age- and gender-matched general population sample
2. The association between depression stigma and
  - the exploration of suicide risk and
  - the consecutive management of suicidal callers.
3. The association between self-rated knowledge and
  - the exploration of suicide risk and
  - the consecutive management of suicidal callers.

For aim 1, we hypothesize that TES counselors report less personal depression stigma compared to the general population sample, as previous studies indicated an association between personal depression stigma and contact with people with depression [10, 11, 32]. We assume that the type of contact a TES counselor has (working in part-time and on a voluntary basis) differs substantially from a professional medical contact.

Objectives 2 and 3 will be analyzed in an explorative manner since there are no comparable studies for that specific sample regarding depression stigma and its impact.

## Methods

### Participants and procedures

#### Sample 1: counselors of the telephone emergency service (TES)

Recruitment took place in all 108 TES centers in Germany in 2012 [33]. All counselors were informed about the anonymous and voluntary online survey. The survey was available for 8 weeks in 2012 and 893 counselors took part, which equals a response rate of 10.5% (total number of counselors working for the TES in Germany in 2012 = 8500). There are no comparable response rates from other TES surveys, nevertheless it can be considered as low and potentially be explained by recruitment related reasons (see discussion section, p. 17, 18). The total sample of 893 participants includes missing data. Therefore, a sensitivity analysis was conducted to compare the total sample with the subgroup of completers (*n* = 704). Since significant differences were found between the two samples (e.g. regarding age and stigma), it can be assumed that missing values are not random. Therefore, the total sample (*N* = 893) will be considered for all further analysis and the number of participants with valid data are reported in the results section instead of imputing missing values.

#### Sample 2: general population sample (OSPI-Europe)

The OSPI-Europe project comprised community samples interviewed via telephone. The baseline data (collected in 2009) of Germany were analyzed. The sample of 1002 participants was representative to the local population in terms of gender and age distribution. A full sample description can be found elsewhere [34].

### Instruments

In both samples, socio-demographic information and attitudes toward depression using the Depression Stigma Scale (DSS [35]) were assessed. The DSS measures personal and perceived stigma with nine items each. The items are scored on a five-point Likert Scale ranging from “strongly disagree” (score 1) to “strongly agree” (score 5). Higher sum scores on each scale and in total indicate more stigmatizing attitudes. The DSS has demonstrated high test-retest reliability and moderate to high internal consistency in different populations (Cronbach’s alpha ranging from .77–.82 for total, personal and perceived stigma scale in a national Australian sample and from .75–.82 in a psychological distressed subset [11], Cronbach’s alpha of .70 and .77 for personal and perceived scale in a sample of adolescents [10]) and across various countries, e.g. Germany, Netherlands and Japan [36–38].

Additionally, the counselors rated their knowledge about depression and suicidality on a 4-point rating scale from “poorly informed” (score 1) to “very well informed” (score 4) and they answered questions regarding exploration of suicide risk and management of suicidal callers. The items (relevant for this analysis) were as follows (response categories): 1) When do you pose concrete questions about suicidality? (In every call vs. If I get suspicious vs. If proof becomes more and more evident throughout the conversation vs. Only if the caller mentions suicidality himself vs. Never). Items 2 and 3 each relate to the last call, where counselors dealt with suicidality: 2) Please think about the last call, that dealt with suicidality: what type of suicidal behavior was mentioned by the client? (Occasional thoughts of suicide vs. Recurrent thoughts of suicide vs. Detailed plan of the suicidal act vs. Active suicidal behavior vs. None vs. I don’t know); 3) How did you react? (Didn’t go into detail vs. Asked for reasons vs. Informed about specific contacts vs. Advice of seeking help immediately vs. Called ambulance/the police vs. Others vs. I don’t know). For the analysis, a caller was considered to be at risk for suicide (suicidal caller) if the counselor answered item 2 as follows: a caller mentioned either recurrent thoughts of suicide, a detailed plan of the suicidal act or active suicidal behavior. The set of items was developed in an interdisciplinary team of psychologists, senior psychiatrists and crisis hotline counselors for the purpose of this study, as no established instruments for this kind of assessment were available.

### Data analysis

Statistical analysis was performed using IBM SPSS Statistics version 24.0. Levels of significance are reported two-sided, with a nominal level of significance set at *p* < .05. For the TES sample, sociodemographic variables were analyzed descriptively using measures of central tendency and portions. To examine subgroup differences depending on self-rated knowledge, chi-square tests for cross tables (nominal data) and Mann-Whitney U tests or Kruskal-Wallis tests (rank-scaled data) were utilized. For post-hoc analysis in case of multiple tests Bonferroni correction was applied. Because of the ordinal scale level of the single DSS items the median and the interquartile range were chosen as descriptive statistics for DS scale scores. For the same reason, nonparametric tests were performed in order to test group differences in DS sum scores. To analyze univariate associations in the TES sample between stigma scores and potential related factors, Spearman-Brown correlations were calculated. In order to test the association between the self-rated knowledge about suicidality and the consecutive management of a suicidal caller for statistical significance based on a 4 × 6 cross table, the exact Fisher-Freeman-Halton test, an extension of Fisher’s exact test for 2 × 2 cross tables, has been applied. The exact *p* value was estimated by using a Monte Carlo simulation after 10,000 iterations. In this context, a 95% confidence interval was given, too.

To compare the TES and the general population sample regarding depression stigma, first a propensity score matching was performed. Based on their propensity scores calculated by logistic regression (nearest neighbor matching algorithm, caliper 0.2 [39]), samples were matched by age and gender. Differences in the association of DS scores and age between TES and the general population sample were investigated by a *r*-to-*z* transformation for independent samples using the online calculator VassarStats.net [40]. Effect sizes were interpreted as suggested by Cohen [41].

## Results

### Sample description

Respondents from the TES and the general population sample significantly differed in gender (χ^2^ = 129.15, *df* = 1, *p* < .001) and age (*Z* = − 11.15, *p* < .001; Table 1). Due to missing values in the total sample of TES counselors (*N* = 893), valid percentages and in columns the number of participants with valid data is reported. The majority of counselors reported not to work in the health sector (79.2%, *N* = 846). Regarding their engagement for the crisis hotline, 35.5% of the participants worked for the hotline for up to 5 years, 25.0% 5 to 10 years and 30.1% (*N* = 893) for more than 10 years. Almost all counselors (91.5%, *N* = 893) have been confronted with suicidality during their work for the TES. According to the counselor’s self-report, every second call (*M* = 49.8%, *SD* = 20%) dealt with depression. The outright majority considered themselves as well or very well informed about depression (85.5%, *N* = 890; *Mdn* = 3, *IQR* = 3–3) and suicidality (84.6%, *N* = 887; *Mdn* = 3, *IQR* = 3–3).

1. Depression stigma in the Telephone Emergency Service (TES) sample (N = 893) and in comparison to an age- and gender-matched general population sample (*N* = 1002)

**Table 1:** Demographic characteristics of counselors of the Telephone Emergency Service (TES) and general population sample

Characteristic	TES (*N* = 893)	General population sample (*N* = 1002)
	*n* (%)	*n* (%)
Gender		
male	238 (26.7)	478 (47.7)
female	655 (73.3)	524 (52.3)
Age categories		
0: < 21	0	18 (1.8)
1: 21–30	12 (1.3)	110 (11.0)
2: 31–40	32 (3.6)	284 (28.3)
3: 41–50	193 (21.6)	158 (15.8)
4: 51–60	352 (39.4)	151 (15.1)
5: 61–70	253 (28.3)	128 (12.8)
6: > 70	51 (5.7)	153 (15.3)
Median age (*IQR*)	category 4 (3–5)	category 3 (2–5)
Marital Status		
Never married	96 (10.8)	158 (15.8)
currently married /cohabiting	622 (69.7)	609 (60.8)
divorced, separated, widowed	160 (17.9)	235 (23.5)
Not specified	15 (1.7)	0
Occupational status		
Student	18 (2.0)	42 (4.2)
Self-employed	114 (12.8)	98 (9.8)
Paid work	366 (41.0)	432 (43.1)
Retired/ House keeping	286 (32.0)	363 (36.2)
Unemployed	19 (2.1)	64 (6.4)
Volunteer work	71 (8.0)	3 (0.3)
Not specified	19 (2.1)	0

*IQR* interquartile range

In total, counselors of the TES scored significantly lower on the personal stigma scale (*Mdn* = 16, *IQR* = 13–18) than on the perceived stigma scale (*Mdn* = 29, *IQR* = 26–33), *Z* = − 25.31, *p* < .001. Investigating the association of years working for the TES and depression stigma revealed a negative, but small effect for stigma sum score (*r*_*s*_ = −.12, *p* < .001), personal stigma score (*r*_*s*_ = −.08, *p* = .026) and perceived stigma score (*r*_*s*_ = −.09, *p* = .006). A significant lower personal stigma score (*Mdn* = 25, *IQR* = 20–30) than perceived stigma score (*Mdn* = 31, *IQR* = 27–35.5; *Z* = − 23.69, *p* < .001) was reported by the participants of the general population sample, too. In the TES sample, a higher stigma sum score and higher perceived stigma score were significantly associated with younger age (*r*_*s*_ = −.10, *p* = .003, *r*_*s*_ = −.11, *p* = .001). On the other hand, participants of the general population sample showed significantly higher stigma sum scores and higher personal stigma scores (*r*_*s*_ = .08, *p* = .011, *r*_*s*_ = .15, *p* < .001) with higher age.

To compare the TES and the general population sample (between group comparisons) regarding depression stigma and the association with age, a propensity score matching was performed (see methods section for details). Due to matching, samples did not differ regarding gender (χ^2^ = .18, *df* = 1, *p* = .675) and age (*Z* = .003, *p* = .973). The matched samples significantly differed in both stigma subscales and in stigma sum scores with high effect sizes for stigma sum score and personal stigma score and a rather low effect size for perceived stigma score. According to our hypothesis, the TES counselors reported significantly lower personal stigma scores. Further, the data suggest that also the sum and perceived stigma scores are lower than in the general population sample (Table 2).

**Table 2:** Differences in depression stigma between Telephone Emergency Service sample and general population sample *(*PS-matched)

Stigma Score	TES Median (*IQR*) *(n = 577)*	General population sample Median (*IQR*) *(n = 577)*	*Z*	Effect size *r*
Personal stigma score	16 (13–18,5)	25 (20–30)	**−20.215**	−.595
Perceived stigma score	29 (26–33)	31 (27–35,5)	**−6.079**	−.179
Stigma sum score	45 (40–50)	56 (49–63)	**−17.708**	−.521

*IQR* Interquartile range, *PS* Propensity score, *r* = *Z*/√*N*; Bolded values indicate *p* < .001

As displayed in Table 3, age and stigma sum score were significantly associated in both matched samples (TES and general population), but in opposite direction: Whereas the stigma sum score was significantly lower in younger subjects of the general population sample, TES counselors showed lower stigma sum scores in higher age groups. The same pattern was found for perceived stigma scores.

2. and 3. Depression stigma and self-rated knowledge in Telephone Emergency Service (TES) sample and association with exploration of suicidality and consecutive management of suicidal callers

**Table 3:** Association of depression stigma and age in Telephone Emergency Service sample and general population sample (PS-matched)

Spearman correlation with age	TES *(n = 577)*	General population sample *(n = 577)*	*Z*
Personal stigma score	.009	**.117**	1.84
Perceived stigma score	**−.120**	.05	**2.89**
Stigma sum score	**−.104**	**.103**	**3.52**

*Z* calculated by *r-to-z-transformation* [31]; *PS* = Propensity Score; Bolded correlations are significant at the .05 level

Self-rated knowledge about depression as well as about suicide showed small negative associations with stigma sum score (*r*_*s*_ = −.14*, p* < .001; *r*_*s*_ = −.13*, p* < .001) and personal stigma score (*r*_*s*_ = −.25, *p* < .001; *r*_*s*_ = −.24, *p* < .001).

When the counselors were asked to report when they explore suicidality, 36.3% answered “If I get suspicious”, 37.6% “If proof becomes more and more evident throughout the conversation” and 25.6% stated to ask only if the caller mentions suicidal ideations. Only 3 counselors noted that they never explore suicidality and one counselor reported to ask in every call (*N* = 893). Depending on their self-rated knowledge about suicidality, counselors significantly differed in posing concrete questions about suicidality (χ^2^ = 73.03, *df* = 12, *p* < .001; see Table 4). Post-hoc analysis applying Mann-Whitney U tests revealed significant differences in the majority of subgroups (data not shown, see Additional file 1).

**Table 4:** Exploring of suicide risk depending on self-rated knowledge in Telephone Emergency Service sample

	Self-rated knowledge about suicidality (*N* = 887)			
	Poorly informed (*n* = 11)	Less well informed (*n* = 126)	Well informed (*n* = 643)	Very well informed (*n* = 107)
When do you pose concrete questions about suicidality? (*N* = 887)	%	%	%	%
Never (*n* = 3)	9.1	0	0.3	0
Only if the caller mentions suicidality himself (*n* = 227)	54.4	33.3	25.2	15.9
If proof becomes more evident throughout conversation (*n* = 334)	9.1	46.8	38.6	24.3
If I get suspicious (*n* = 322)	27.3	19.8	35.8	59.8
In every call (*n* = 1)	0	0	0.2	0

**Fig. 1:**
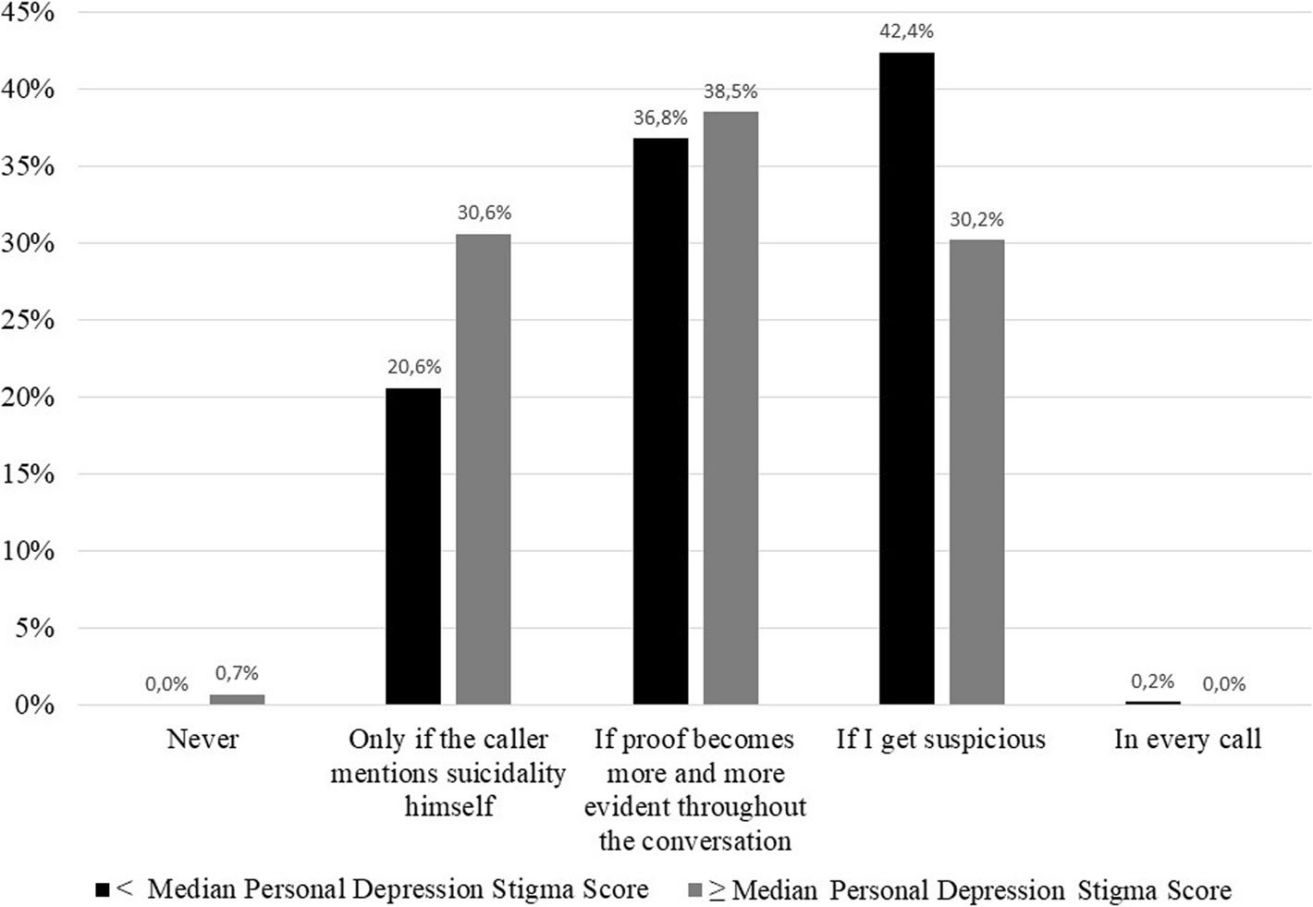
Association of exploring suicidality and personal depression stigma score (split by median of scores)

The most frequently reported way by the counselors to manage a caller at risk for suicide (reported in the last call; *N* = 557) with 59.2% was “Asking for reasons for suicidality”, followed by “Informed about specific contacts” (14.9%), “Advice of seeking help immediately” (10.4%), “Others” (11,3%), “Called an ambulance/the police” (3.6%) and “Didn’t go into detail” (0.5%). The association between the self-rated knowledge about suicidality and self-rated management of a caller at risk for suicidality failed to be statistically significant (Fisher-Freeman-Halton test: Monte Carlo *p* value (95% CI) after 10,000 iterations: *p* = 0.44 (0.43–0.45)). Investigating the relationship of depression stigma and exploring suicidal callers, counselors with personal stigma scores below the median of 16 (*n* = 446) differed significantly from those with personal stigma scores above median (*n* = 447), *Z* = − 4.46, *p* < .001, *r* = .15. This association was not found for perceived stigma score. A personal stigma score below the median was associated with exploring callers as soon as the counselor suspects risk for suicidality (42.4% vs. 30.2% for counselors with personal stigma score above the median; see Fig. 1). Counselors with a personal stigma score above median reported to explore, only if the caller mentions suicidality himself compared to counselors with personal stigma score below median (30.6% vs. 20.6%).

Counselors with personal stigma score below the median did not differ from counselors with personal stigma score above the median regarding their reported management of callers at risk for suicide (Fisher-Freeman-Halton test: Monte Carlo *p* value (95% CI) after 10,000 iterations: *p* = 0.96 (0.96–0.96)). For perceived stigma scores no differences could be detected.

## Discussion

### Depression stigma in the telephone emergency service (TES) sample and in comparison to an age- and gender-matched general population sample

This is to our knowledge the first study investigating depression stigma in a sample of crisis hotline counselors that reported in comparison to a representative general population more favorable attitudes toward depression with large effects for overall and personal stigma and a small effect for perceived stigma. This result are according to hypothesis 1 and can be considered as overall positive given the fact that the counselors of the TES reported to deal with depression in every second call and stigmatizing attitudes toward mental illness potentially negatively impact e.g. the quality of health service like health care decision or referring to specialist care, which has been investigated for mental health and primary care providers dealing with mental disorders [6, 12].

Moreover, the TES considers itself as a low-threshold service guaranteeing anonymity and confidentiality and hereby especially reaching out to people that avoid seeking professional help due to several reasons, e.g. being suspicious or afraid of potentially active rescue procedures as well as stigmatization [19]. Therefore, less depression stigma on the side of the counselors potentially plays an important role regarding the target population of people seeking anonymous help and advice in crisis situations.

A possible explanation for the lower personal depression stigma in comparison to the general population sample could be the engagement for the TES itself, and not working in the health care sector (as main job), which is the case only for 20% of the counselors. This is further supported by a small negative association between depression stigma and the duration of working for the TES. In like manner, previous studies showed a lower personal depression stigma being associated with higher levels of contact to people with depression [10, 32]. This is being explained by first-hand-experiences yielding in higher understanding and more tolerance, whilst working for the TES can be considered as first-hand experiences, given the high prevalence of reported psychiatric diagnoses in callers of crisis lines across different services and countries (e.g. [22]). Also, the training the counselors receive could explain this result and is in line with previous research demonstrating an association between knowledge (different assessments) and depression stigma [11, 32]. A self-selection of persons working voluntarily for crisis support services might also explain the stigma differences between the counselor sample and the general population sample. Previous research demonstrated differences in personality characteristics between crisis hotline volunteers and non-volunteers in empathy factors and agreeableness (sample of students [42]), in tolerance and psychological mindedness (crisis hotline counselors vs. matched control [43]) and differences in prosocial motivation explained by interpersonal values like harmony and helpful influence (sample of TES volunteers compared to matched nonclinical reference sample [44]). It is also conceivable that a combination of both - self-selection as well as the engagement for the TES - explain differences in the two samples and in depression stigma scores.

Both samples scored significantly lower in personal depression stigma than in perceived depression stigma which is consistent with other studies [37, 45] and might be attributed to social desirability aspects or a general overestimation of stigma in society [10].

Analyzing the association of age and depression stigma in the matched samples revealed that older crisis hotline counselors reported less overall depression stigma compared to older participants of the general population sample, showing more overall depression stigma. The same association is being found for perceived stigma depression stigma between the two matched samples. Within the (matched) samples, counselors of higher age reported less overall depression stigma than younger counselors. Within the general population sample, the association was inverse. A secondary analysis showed, that in the total, unmatched samples, the results are comparable. Previous research on demographic factors as possible predictors of depression stigma showed inconsistent findings for age [10, 32, 34] and most of these studies were cross-sectional, not allowing any causal inferences [46]. The age effects for the TES counselors found in the current study could be interpreted in line with results from a longitudinal study on social distance (as a measure of discriminatory attitudes) toward people with depression, postulating that attitudes over the life span do change depending on personal experiences [46].

### Depression stigma and self-rated knowledge in telephone emergency service (TES) sample and association with exploration of suicide risk and consecutive management of suicidal callers

More than 90% of the counselors stated experiences with suicidality reported by callers. One quarter of the counselors (26%) answered in the survey that they never pose concrete questions about suicide risk or only if the caller mentions suicidality himself. It is important to mention that the assessment or exploration of suicide risk is not defined as an obligation in the policy of TES, but it seems to be an essential part of the practical routine (based on self-report). Other studies, using different methodological approaches, like silent monitoring of calls and post-hoc external ratings or surveying callers retrospectively revealed rates of risk assessment between 50 to 60%, whilst risk assessment had been mandatory by the policy of the respective service [17, 22].

Self-rated knowledge about suicide and personal depression stigma were associated with exploring suicide risk as follows: Despite already comparably low personal depression stigma in the TES sample, counselors reporting less personal stigma (median split) or having rated themselves as very well informed about suicidality explored suicide risk more actively. They rather pose concrete questions about suicidality as soon as they get suspicious instead of only if the caller mentioned suicidality himself. However, the self-rated management of a caller at risk for suicide was neither associated with depression stigma of a counselor, nor with self-rated knowledge about suicidality. In case of a caller being at risk for suicide, none of the counselors reported to do nothing. Less than one fifth of the counselors reported to advice seeking help immediately or to inform a caller at risk about specific contacts for professional help. By far the most frequent answer regarding the management of a suicidal caller was asking him for reasons for suicidal ideation. Several studies on counselors behavior and intervention styles highlighted the role of a good contact between counselor and caller [14, 23, 47]: Good contact includes amongst others especially empathy and respect, active engagement with a suicidal caller, and comprising the discussion of thoughts of suicide- which then contributes to a decrease of depressive mood and crisis status.

After all, one of the most important evaluation a crisis hotline counselor has to make is to determine whether a caller is at risk for suicide (as a symptom of a psychiatric disorder or a suicidal crisis not meriting a psychiatric diagnosis [13]) and therefore in need of an emergency intervention [48]. The present study reveals factors being associated with this important exploration, that should be considered e.g. in training of counselors, also in future research in this field.

### Strength and limitation

To our knowledge this is the first study on depression stigma of crisis hotline counselors. Moreover, the German TES “Telefonseelsorge” is the largest and major telephone crisis service in Germany. The study may have the following limitations: the response rate was comparably low, which might be due to the recruitment strategy. The TES service centers were informed about the survey via their umbrella organization. There is no information to what extent they replied the request to inform their volunteers on the survey and in which way. Therefore, the survey was possibly not sufficiently known in all TES service centers. A selection bias cannot be ruled out, as the survey was on a voluntary basis. This may lead to an overrepresentation of well-informed and motivated TES counselors in the survey. Also, an underrepresentation of counselors with a higher depression stigma is possible (refusing to participate e.g. due to fearing consequences for their engagement, despite guaranteed anonymity and avoiding the term “stigma” in the introduction of the survey). Due to this potential bias, the representativeness of this sample for all TES crisis hotline counselors is not determinable.

Additionally, only self-report measures had been applied and the assessment of the management of suicidal callers reported by the counselor via self-report does not necessarily display the actual behavior. All items (besides the DSS) were developed in a focus group approach for the specific purpose of this study and are therefore not validated so far. At the time of designing this study, there were no for this specific sample suitable measures which could have been applied or adapted for this study. Furthermore, we followed the recommendation of the IRB of the Protestant and Catholic conference for telephone pastoral care to minimize the effort for the participants by applying a minimum number of items.

Social desirability might be present as well, which is a major problem stigma research generally deals with. Further, not all participants answered all questions and finally, the survey was cross-sectional, so no causal inferences can be drawn.

## Conclusion

Counselors working for the TES did show fewer stigmatizing attitudes toward depression compared to a general population sample. Further, the results of the current study suggest that favorable attitudes toward depression as well as feeling informed about suicidality go along with more confidence in exploring risk for suicide; even they do not seem to influence the management of a suicidal caller. The results indicate that a profound training, addressing potential mental health stigma and hands-on information about depression and suicidality seem to be essential for volunteer counselors working for crisis hotlines, at the beginning and also on a regular basis during their engagement.

## Supplementary information

**Additional file 1.** Post-hoc analysis of self-rated knowledge about suicidality and differences in exploring of suicidality.

## Data Availability

The datasets analyzed during the current study are available from the corresponding author upon reasonable request.

## Abbreviations

DSS: Depression stigma scale
IQR: Interquartil range
TES: Telephon Emergency Service
