# Supplementary material for: Depression stigma and management of suicidal callers: a cross-sectional survey of crisis hotline counselors
Source: BMC Psychiatry. 2019 Nov 6;19:342. doi: 10.1186/s12888-019-2325-y (PMC6836490; doi:10.1186/s12888-019-2325-y)
Supplement: Supplementary file 1 — Additional file 1. Post-hoc analysis of self-rated knowledge about suicidality and differences in exploring of suicidality. [file 12888_2019_2325_MOESM1_ESM.docx]

**Additional file 1**

**Titel:** Post-hoc analysis of self-rated knowledge about suicidality and exploring of suicidality

**Description of data:** Differences in exploring suicidality between subgroups of self-rated knowledge about suicidality (Z-Value)

| Self-rated knowledge on suicidality | Poorly informed | Less well informed | Well informed | Very well informed |
| --- | --- | --- | --- | --- |
| Poorly informed | − | -1.32 | -2.00 | **-2.93** |
| Less well informed |  | − | **-3.20** | **-5.70** |
| Well informed |  |  | − | **-4.23** |
| Very well informed |  |  |  | − |

Mann-Whitney U test with Bonferroni correction of alpha = .05 / 6 = 0.0083;

Bolded values indicate *p* < .01.
